# Multiple Self-Healing Squamous Epithelioma (MSSE): A Digenic Trait Associated with Loss of Function Mutations in *TGFBR1* and Variants at a Second Linked Locus on the Long Arm of Chromosome 9

**DOI:** 10.3390/genes11121410

**Published:** 2020-11-26

**Authors:** David Goudie

**Affiliations:** Regional Genetics Service, NHS Tayside, Dundee DD1 9SY, UK; david.goudie@nhs.scot

**Keywords:** multiple self-healing squamous epithelioma, MSSE, *TGFBR1*, Loeys Dietz syndrome

## Abstract

MSSE (Ferguson-Smith disease) is a rare familial condition in which multiple skin tumors resembling squamous carcinomas invade locally and then regress spontaneously after several months, leaving disfiguring scars. We review evidence from haplotype studies in MSSE families with common ancestry that the condition is caused by loss of function mutations in *TGFBR1* interacting with permissive variants at a second linked locus on the long arm of chromosome 9. The spectrum of *TGFBR1* mutations in MSSE and the allelic disorder Loeys Dietz syndrome (characterized by developmental anomalies and thoracic aortic aneurysms) differ. Reports of patients with both MSSE and Loeys Dietz syndrome are consistent with variants at a second locus determining whether self-healing epitheliomas occur in patients with the loss of function mutations found in most MSSE patients or the missense mutations in the intracellular kinase domain of *TGFBR1* that characterize Loeys Dietz syndrome.

## 1. Clinical Features

Multiple self-healing squamous epithelioma (MSSE, OMIM 132800) is a rare familial form of skin cancer characterized by multiple skin tumors of the face and limbs. These lesions heal spontaneously after several months leaving characteristic pitted scars if they are not excised.

MSSE was first described in a 23 years old Scottish coal miner [1]. MSSE can be variable in its severity and age of onset but his history is typical of many patients. He developed multiple lesions on his legs aged 16. Each tumor started “at first as a reddish macule, became papular, enlarged, ulcerated and ultimately healed leaving pitted scars. The individual lesions lasted for months, and fresh ones appeared in numbers rather more than sufficient to balance the healing so that the count of active lesions slowly increased. His face and ears soon became affected, and one or two lesions appeared on his arms and thighs”.

**Figure 1:**
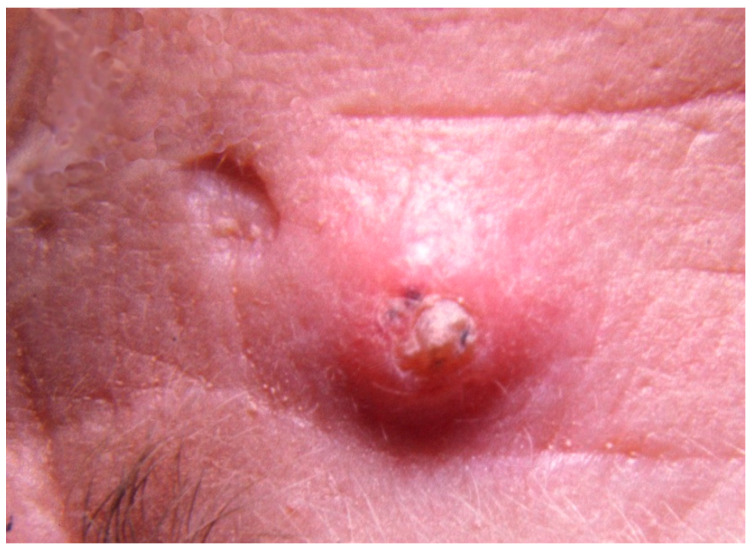
A small (5 mm) self-healing epithelioma with a central keratin plug adjacent to a pitted scar at the site of a lesion that resolved spontaneously.

The condition can be very disfiguring in severely affected individuals. Some affected individuals have one or two tumors during their lifetime while others have had over 100 primary skin tumors [2]. The age of onset of a first tumor is variable ranging from 8 to over 70 years (median 28 years). As each tumor involutes it forms a horny plug that falls out often leaving a deep pit with irregular, over-hanging edges (Figure 1). Lesions on the limbs are typically larger than those on the face, scalp and ears, and leave flat scars. Tumors arise most frequently on the sun-exposed skin of the head, neck and limbs. The nose, ears and circumoral regions are most frequently affected. The trunk is seldom affected. Palmar and plantar skin are not affected suggesting that the tumors may arise from hair follicles [2].

**Figure 2:**
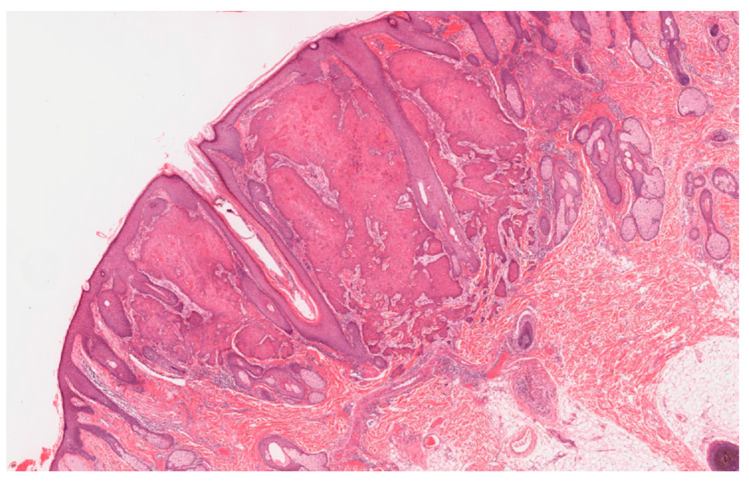
A multiple self-healing squamous epithelioma (MSSE) tumor that has the histological appearance of a well differentiated squamous cell carcinoma with invasion of the reticular dermis.

**Figure 3:**
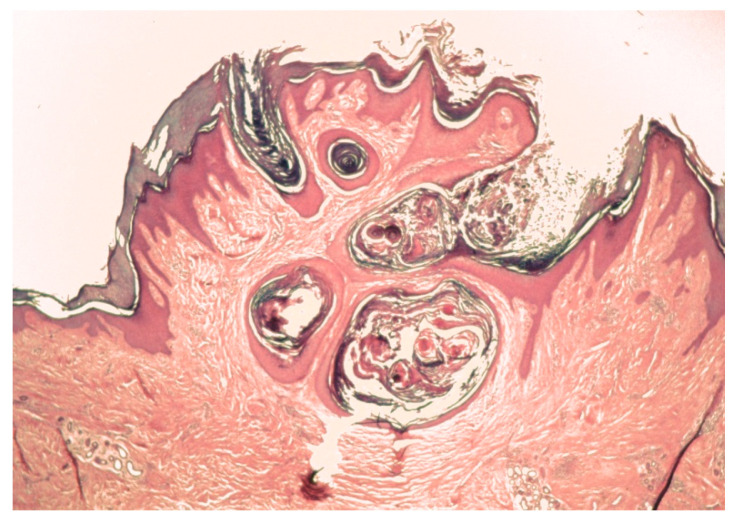
An involuting tumor with cornified “nests” coalescing into a keratin plug in the dermis.

The MSSE tumors have a similar histological appearance to well-differentiated squamous carcinomas, with invasion deep into the dermis. Lymphocytes often infiltrate the tumors but their role in tumor resolution is not known. Invasive epithelial columns show central keratinization with the formation of cornified ‘nests’, which as the tumor involutes, coalesce into a horny plug (Figure 2 and Figure 3). The base of the resolving lesion shows marked fibrosis of the dermis with loss of elastic fibers. The skin tumors follow a similar clinical course to sporadic keratoacanthomas. MSSE tumors can usually be distinguished from keratoacanthomas because of the occurrence of multiple lesions, the earlier ages at which tumors develop and the familial nature of the condition. The diagnosis of MSSE can be confirmed by detection of constitutional *TGFBR1* mutations (see below). Although the tumors are invasive and can be locally destructive, distant metastases have not been reported.

Tumors are most commonly treated with local surgical excision but many patients avoid treatment and prefer to await spontaneous regression despite the scars that can result [3]. Etretinate therapy has been reported to reduce tumor numbers in some patients [4,5]. Treatment with radiotherapy must be avoided because more numerous, more invasive tumors arise in previously irradiated skin [3,5,6].

In a study of 11 independently ascertained Scottish families including 62 affected family members, MSSE was found to segregate as an autosomal dominant trait [2]. This study was undertaken by Professor Malcom Ferguson-Smith, a clinical geneticist and the son of John Ferguson-Smith, the dermatologist who first described the condition. Of the 74 offspring of obligate carriers, 37 were affected and 37 unaffected; among affected offspring 19 were male and 18 female.

## 2. Genetic Linkage

Linkage studies using polymorphic DNA markers in 11 affected families (41 affected individuals and 110 of their first degree relatives) showed that MSSE segregates with markers for the long arm of chromosome 9 at 9q22.3q31 [7]. The MSSE locus was tightly linked to the polymorphic DNA marker D9S53 with a maximum lod score of 9.02 at a recombination fraction 0.03.

Ten families were from Scotland and one was from the north east of England. Comparison of marker alleles segregating with MSSE revealed that the same haplotype of 9q22.3 markers was segregating with MSSE in 9 of these families [7,8,9] (Table 1). Four branches of the largest family (called Family GE in Reference [7] and Family 4 in Reference [9] were originally ascertained independently. Genealogical studies [2] revealed that the affected members of this family were all descended from a couple that married in west central Scotland in 1810. The affected members of the 9 families must have inherited the shared haplotype from a common ancestor that lived over 200 years ago.

There was a surprising lack of recombination events in the shared haplotype. The haplotype has been inherited intact through many generations [7,8,10]. This lack of recombination precluded precise localization of the genetic variants predisposing to MSSE. The haplotype shared by 38 affected members of 7 families (families 4–10 in Table 1) spans about 9.9 Mb of the long arm of chromosome 9 and includes at least 138 genes. Haplotype comparisons revealed evidence for recombination events in previous generations of two affected families. Affected members of families 2 and 18 (Table 1) only shared part of the haplotype spanning 4 Mb of chromosome 9 centromeric to the XPAC gene, indicating that the MSSE gene was likely to lie in this 4 Mb region [8,9].

## 3. Pathogenic *TGFBR1* Sequence Variants Cause MSSE

Efforts to identify the disease gene with Sanger sequencing of many of the genes within the conserved 9q22.3 haplotype were unsuccessful [11]. The advent of high-throughput massively parallel sequencing made it possible to screen for pathogenic sequence variants in many more genes.

Exon array capture of coding sequences and high-throughput genomic sequencing from a 24 Mb region of chromosome 9 that included the whole of the 9.9 Mb haplotype shared by most affected Scottish families’ identified independent mutations in the transforming growth factor beta receptor gene, *TGFBR1*, in three unrelated families [9]. Subsequent dideoxy sequencing of *TGFBR1* identified 11 distinct monoallelic mutations in 18 affected families, firmly establishing *TGFBR1* as the causative gene. *TGFBR1* mutations were found in all 67 affected individuals that were genotyped from the 18 families with mutations. The age of onset of a first tumor in mutation carriers was between 8 and 81 years. Sixteen asymptomatic mutation carriers were found (mean age 36.5 years, range 19–79 years).

**Figure 4:**
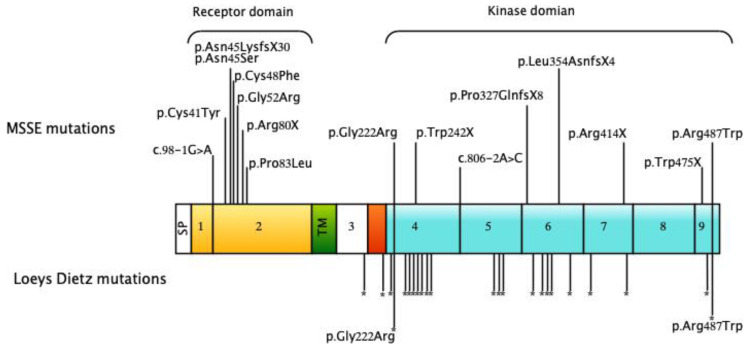
Mutations in *TGFBR1* in individuals with MSSE (modified from Reference [9] Domain structure of the TGFβRI protein showing reported MSSE mutations [6,9,12,13,14,15,16] and unpublished data). The MSSE mutations are shown above the protein while the locations of missense mutations reported in Loeys Dietz syndrome [17,18,19,20,21,22,23,24,25,26,27,28,29] are indicated by asterisks below. The location of the c.1459C > T, p.(Arg487Trp) and c.664G > A, p.(Gly222Arg) variants found in patients with both MSSE and Loeys Dietz phenotypes are also shown. Functional domains are color-coded: extracellular ligand-binding domain (yellow), transmembrane domain (green), glycine-serine-rich domain (red), cytoplasmic kinase domain (blue). SP, signal peptide; TM, transmembrane domain.

The pathogenic sequence variants included mutations in the extracellular ligand-binding domain and a series of truncating mutations (Figure 4). Since then, *TGFBR1* mutations have been reported in 9 other families in the literature [6,12,13,14,15,16] or found through diagnostic testing (unpublished data). These variants together with variants described in Reference [9] are shown in Figure 4. The c.122G > A p.(Arg80X) variant has been found in three independently ascertained patients from New Zealand [9], Switzerland [16], and the UK [6]. The frameshift variant c.980delC p. (Pro327GlnfsX8) variant has been found in three Scottish families and the frameshift variant c.1059_1062del 6 ACTGinsCAATAA p.(Leu354AsnfsX4) variant has been found in two families in Scotland.

No pathogenic *TGFBR1* sequence variants or larger scale deletions or duplications of the *TGFBR1* gene were detected in affected members of four families with familial self-healing skin tumors [9]. The etiology of the skin tumors in these families is unknown.

Analysis of DNA from micro-dissected tumors from MSSE patients with constitutional *TGFBR1* mutations shows somatic loss of heterozygosity at the *TGFBR1* locus with retention of the mutant allele consistent with *TGFBR1* behaving as a tumor suppressor gene [9,10]. Constitutional heterozygous loss of function *TGFBR1* mutations do not appear to have any effect on development, suggesting that transforming growth factor beta (TGFβ) signaling is not seriously perturbed in most cells with a single functional copy of the *TGFBR1* gene. Tumors develop from skin keratinocytes where a “second hit” has resulted in loss of the remaining functional *TGFBR1* allele.

In a Tgfbr1 mouse model [30] carrying a p.Y378 * nonsense mutation heterozygous mutant mice were morphologically indistinguishable from wild type mice. In this model, the mutant allele is degraded by nonsense mediated mRNA decay. Homozygous mutant mice die during embryogenesis due to defective vascularization. A macroscopic evaluation of the skin of fifteen 12-month-old heterozygous Tgfbr1 mutant mice did not reveal any scars similar to those observed in human MSSE patients after spontaneous regression of the skin tumors.

## 4. Digenic Inheritance: *TGFBR1* and a Second Linked Locus on the Long Arm of Chromosome 9

The finding that *TGFBR1* is the MSSE gene was unexpected because the *TGFBR1* gene lies outside the 4 Mb haplotype shared by all nine Scottish families with common ancestry (Table 1). It does, however, lie within the 9.9 Mb haplotype shared by seven of the nine families [10]. The same *TGFBR1* mutation p.(G52R) was found in all seven families with the larger haplotype. The two “recombinant” families (families 2 and 18) that share only the shorter 4 Mb haplotype that does not extend to *TGFBR1* have different *TGFBR1* mutations (p.(N45S) and p.(R414X)). These findings indicate that a second locus, within that part of the Scottish haplotype shared by all nine families, including families 2 and 18, is required for MSSE pathogenesis as it is very unlikely that all the families share the same rare haplotype by chance. The rarity of this 4 Mb disease associated haplotype was confirmed by the identification of nine additional rare sequence variants in the introns of five genes within the haplotype in targeted sequencing studies in members of the Scottish families including families 2 and 18 [31]. In 231 unrelated healthy controls including 118 Scottish individuals, the minor allele frequency of the nine variants ranged from 0 to 0.022.

The shared haplotype segregates with MSSE in families with missense variants in the extracellular domain of the *TGFBR1* and a family with a truncating nonsense mutation. The haplotype has not been found in several other Scottish families [8] or in any families that are not of Scottish descent [11]. Despite this, it is likely that the etiology of the condition is the same in all families with MSSE caused by mutations in *TGFBR1*. Permissive variants at the second locus that have arisen independently in unrelated families will not be associated with shared haplotypes.

The evidence for digenic inheritance is strengthened by the remarkable lack of recombination within the extended 9 Mb haplotype shared by all affected members of seven families. If mutations in *TGFBR1*, which lies at the telomeric end of the haplotype, and rare variants at the centromeric end of the haplotype are required for tumor development, ascertainment of affected individuals will select for the intact haplotype. The condition is inherited as a high penetrance dominant trait because permissive variants in *TGFBR1* and the second linked locus are usually inherited together in affected families.

Structural chromosomal abnormalities, such as paracentric inversions, can cause local suppression of recombination but we have observed recombination within the Scottish haplotype in two unaffected family members with an affected parent indicating that it is unlikely that recombination is being suppressed by a structural chromosomal abnormality (unpublished data).

Haplotype comparisons suggest the second locus lies proximal to the XPAC gene in 9q22.3 over 1.5 Mb from *TGFBR1*. As many of the MSSE *TGFBR1* mutations are predicted to abolish expression of *TGFBR1*, it is unlikely that the second locus is a *TGFBR1* cis-regulatory element.

The second locus has not been identified with sequencing coding and non-coding sequences within the shared 4 Mb haplotype but coverage has been incomplete [10,11,18].

## 5. MSSE Is Allelic to Loeys Dietz Syndrome Caused by *TGFBR1* Mutations

Loeys Dietz syndrome is an autosomal dominant aortic aneurysm syndrome. The most typical clinical signs include hypertelorism (widely spaced eyes), bifid uvula or cleft palate and aortic aneurysm with increased tortuosity of the aorta and other major blood vessels. There is a substantial risk of aortic dissection and aneurysms in other arteries [17]. Loeys Dietz syndrome can be caused by mutations in *TGFBR1* [18] and several other genes encoding for components of the TGFβ signaling pathway including *TGFBR2* [17,18,19,20], *SMAD3* [32], *TGFB2* [33,34] and *TGFB3* [35,36].

The spectrum of *TGFBR1* mutations in patients with Loeys Dietz syndrome [17,18,19,20,21,22,23,24,25,26,28,29,37] and patients with MSSE is different (Figure 4). The great majority of *TGFBR1* mutations in patients with Loeys Dietz syndrome are missense variants clustered in or near the region of the gene that encodes the cytoplasmic serine/threonine kinase domain. In-frame deletions in the kinase domain and duplications have also been reported. In contrast, most of the mutations that have been found in MSSE patients are truncating variants distributed throughout the gene or missense variants in the region of the gene encoding the ligand-binding extracellular domain. Two of the truncating mutations have been confirmed to abolish expression of the mutant mRNA in EBV-transformed lymphocytes from MSSE patients [9]. *TGFBR1* mRNA expression associated with other truncating sequence variants has not been studied but the other variants also lie in regions of the gene where they would be expected to promote nonsense mediated decay of mutant mRNA. TGFβR1 protein (extracellular domain) has been detected immunohistochemically in tumors from two affected individuals (one had a splice junction mutation (c.806-2A > C) and the other an extracellular domain missense mutation (p.Gly52Arg)) indicating that in these tumors the mutant proteins were translated and localized to the plasma membrane. If, as these observations suggest, the mRNA product of the *TGFBR1* c.806-2A > C splice acceptor variant is not subject to nonsense mediated decay, the mutant protein would be predicted to lack large parts of the kinase domain abrogating signaling (see below).

Splice junction mutations have been described in patients with MSSE and Loeys Dietz syndrome. Fujiwara and colleagues [38] compared the effects on differential mRNA splicing of a splice donor site variant in *TGFBR1* (c.973 + 1G > A) found in a Japanese patient with familial Loeys Dietz syndrome with the effects of the splice acceptor site variant (c.806-2A > C) discussed above. Ex vivo splicing and functional assays performed in mammalian cells showed that the Loeys Dietz variant generated two types of in-frame transcription products producing functionally inactive proteins whereas the MSSE variant produced an out-of-frame transcript. These results are consistent with the distribution of other mutations, suggesting that dominant-negative variants in the serine threonine kinase domain of *TGFBR1* cause Loeys Dietz syndrome whereas truncating variants cause MSSE.

In silico modeling of the extracellular domain of the TGFβ signaling complex shows that most missense variants found in MSSE patients result in amino-acid substitutions affecting residues that lie along the interface at which TGFβRI interacts with the previously assembled TGFβRII and TGFβ or at the interface at which TGFβRI interacts TGFβ directly [9]. These variants may therefore interfere with recruitment of mutated TGFβRI to the receptor complex.

We know of over 80 patients with MSSE carrying truncating mutations in *TGFBR1* or missense mutations in the ligand-binding extracellular domain of the gene. None of these patients have been reported to have thoracic aortic aneurysms. The *TGFBR1* mutations, found in most patients with MSSE, are not associated with a high penetrance predisposition to the development of aortic aneurysms.

Heterozygous loss of function mutations found in MSSE families do not cause Loeys Dietz syndrome. This is confirmed by studies in mice in which heterozygotes for null and missense *TGFBR1* mutations have been produced [30,39]. Only the mice with a *TGFBR1* missense mutation in the cytoplasmic serine/threonine kinase domain had the Loeys Dietz phenotype [39].

The mechanism by which missense kinase domain *TGFBR1* mutations cause Loeys Dietz syndrome is complex and incompletely understood. *TGFBR1* mutations associated with Loeys Dietz syndrome were found to be inactivating for canonical TGFβ signaling in co-transfection studies with a luciferase reporter or EGFP-tagged SMAD2 in HEK293 cells [40]. Heterozygous mutant cells from transgenic mice had diminished signaling in response to exogenous TGFβ in vitro and they maintained normal levels of Smad2 phosphorylation under steady-state culture conditions, suggesting a chronic compensation. Analysis of TGFβ signaling in the aortic wall in vivo revealed progressive upregulation of Smad2 phosphorylation and TGFβ target gene output, which paralleled worsening of aneurysm pathology and coincided with upregulation of TGFβ1 ligand expression [38].

## 6. *TGFBR1* Variants in MSSE Patients with Aortic Aneurysms

Since *TGFBR1* mutations were first described in patients with MSSE, two patients with MSSE and aortic aneurysms have been described. Both patients carry missense sequence variants in the region of the gene encoding the cytoplasmic serine/threonine kinase domain. The location of these variants is shown in Figure 4.

A 50-year-old woman with MSSE was found to carry a missense mutation in the serine kinase domain of *TGFBR1*, previously reported only in Loeys Dietz syndrome (c.1459C > T, p.Arg487Trp) [14]. She had five confirmed keratoacanthoma-like skin lesions with the first lesion occurring when she was 30 years old. She had one first-degree relative with MSSE. She had mild hypertelorism and a broad uvula consistent with Loeys Dietz syndrome. Computed tomography angiogram demonstrated focal aneurysms of the internal carotid arteries and widespread arterial tortuosity.

A 56 year old man with a five year history of keratoacanthomas over his arms, face and scalp was found to be carrying a *TGFBR1* missense variant (c.664G > A, p.(Gly222Arg)), resulting in substitution of a glycine residue by an arginine at position 222 within the serine/threonine kinase domain [15]. At least seven skin tumors had been excised and diagnosed histologically and he estimated that a further 30 had resolved without treatment. He had an aortic root and valve replacement for aortic regurgitation and aortic root dilatation diagnosed when he was 46 years old. The *TGFBR1* c.664G > A, p.(Gly222Arg)) variant is classified as a variant of uncertain significance using the American College of Medical Genetics and Genomics (ACMG) and the Association for Molecular Pathology (AMP) standards and guidelines for the interpretation of sequence variants [41] but moderate evidence of pathogenicity is provided by its location in a mutational hot spot and multiple lines of computational evidence suggest that the amino acid substitution will have a deleterious effect.

Neither of these patients carry the “at risk” haplotype shared by most MSSE families with Scottish ancestry but these findings are consistent with a requirement for inherited permissive variants at a second locus for the development of tumors in MSSE patients.

## 7. The Second Locus and the Distinctive Spectrum of *TGFBR1* Mutations in Loeys Diets Syndrome and MSSE

If uncommon inherited variants at a second locus are required for development of tumors in *TGFBR1* mutation carriers then only a small proportion of patients with *TGFBR1* mutations ascertained because they have Loeys Dietz syndrome would be expected to have MSSE. Few of them would have inherited a permissive variant at the second locus.

All patients ascertained with MSSE would be expected to have permissive variants at the second locus and so the frequency of the different classes of pathogenic *TGFBR1* variants in these patients should reflect their frequency in the general population. It is likely that the *TGFBR1* variants that predispose to Loeys Dietz syndrome are less common than other classes of pathogenic variants in the gene. The reproductive fitness of carriers of *TGFBR1* variants that cause Loeys Dietz syndrome is likely to be reduced because of the substantial risk of early death from aortic dissection and other vascular complications of the condition.

Pathogenic *TGFBR1* sequence variants are rare and so estimation of the relative frequency of subclasses of these variants in the general population is difficult, even with the large human variant datasets that are now available [42].

## 8. Keratoacanthomas/Squamous Cell Carcinomas in Patients Treated with an TGFβ Monoclonal Antibody

In a phase 1 study using GC1008 (Fresolimumab), a human anti-TGFβ monoclonal antibody, to treat advanced malignant melanoma or renal cell carcinoma, 4 out of 29 patients developed keratoacanthoma or squamous cell carcinoma-like skin tumors [43]. Multiple skin tumors developed. The lesions that were not removed resolved spontaneously over a period of weeks or months after treatment was discontinued. The skin tumors occurred in patients with greater GC1008 exposure, patients treated at higher doses, receiving extended treatment, or both.

A higher proportion of patients (14%) developed skin tumors than would be expected if they had a predisposition to tumor development conferred by variants at the *TGFBR1* linked locus that predispose to MSSE.

In MSSE loss of heterozygosity, studies suggest that tumors arise from cells with biallelic inactivation of *TGFBR1* in a microenvironment with stromal cells with mono-allelic *TGFBR1* mutations. Anti-TGFβ monoclonal antibodies seem to induce development of similar tumors in the absence of permissive variants at the second MSSE locus. Anti-TGFβ monoclonal antibodies may have a more significant effect on TGFβ signaling in stromal cells than inherited mono-allelic *TGFBR1* mutations. Anti-TGFβ monoclonal antibodies may also inhibit signaling in tumor keratinocytes through pathways that do not require TGFβR1.

## 9. Conclusions

Identification of *TGFBR1* mutations in MSSE patients has had some clinical benefits. MSSE can be diagnosed earlier and more reliably, resulting in improved treatment with avoidance of potentially damaging radiotherapy. Testing for pathogenic *TGFBR1* variants can help distinguish between MSSE and conditions such Lynch syndrome in which multiple keratoacanthomas can occur. Evidence from the spectrum of *TGFBR1* mutations in MSSE shows that truncating mutations detected in patients with thoracic aortic aneurysms in the course of diagnostic testing must be viewed with caution and not used as the basis of predictive tests for relatives.

TGFβ signaling has diverse effects on the behavior of epithelial tumor cells and the many stromal cell types that generate and condition the microenvironment in which tumors develop [44]. The mechanisms by which inherited *TGFBR1* mutations cause invasive tumors that consistently resolve spontaneously have still to be elucidated. Whether tumor resolution is due to immunological mechanisms or to intrinsic properties, the tumor cells or their microenvironment is unknown. TGFβ1 exhibits biphasic actions in murine skin squamous cell carcinomas: suppressing early tumor growth but enhancing malignant conversion. It is tempting to speculate that the *TGFBR1* mutations in MSSE have a reciprocal effect, promoting the early stages of tumor development [45]. Identification of the second *TGFBR1* linked locus is likely to improve our understanding of pathogenesis of tumors in MSSE and related sporadic tumors. Somatic *TGFBR1* mutations are common in sporadic squamous cell carcinomas of the skin [46]. In a recent study of squamous cell carcinomas of the skin combining single cell RNA-seq with other methods providing spatial data, it was possible to define tumor and stomal cell populations and the communication network that they engage in cancer [47]. Application of these technologies to tumors from MSSE patients would be likely to yield fascinating results.

## Figures and Tables

**Table 1:** Shared haplotypes in Scottish families with common ancestry (modified from Supplementary Material of Reference [9].

Marker loci	Position	Families		
		4–10	2	18
	Assembly GRCh37/hg19	Alleles		
D9S119	95867389-95867529	1	1	1
D9S197	95875618-95875968	2	2/4 *	2
D9S196	95906010-95906446	5	4/5 *	5
ZNF169CA	96264697-96265065	8	8	8
FBP1 (GA)	96474007-96474379	2	2	1/2 *
AFM070xb11	97021578-97065291	1	1	1
AFM086yf1	97365423-97402531	1	1	1
D9S280	97365423-97344962	2	2	2
FANCC CA Intr2	97912205-98079991	2/3 *	2	2/4 *
FANCC EcoRI	97912205-98079991	2	1/2 *	2
FANCC CA Intr1	97912205-98079991	1	1	1
AFM203WH8 *	98073744-98074099	1	1	1
PTC INT5	98205266-98270831	T	T	T
PTC INT9	98205266-98270831	C	C	C
PTC INT10	98205266-98270831	G	G	G
D9S1816	98276797-98277080	5	5	5/8 *
D9S287	98466090-98466414	3	3	3
AFMa350xgi	98544811-98545188	2	2	2
D9S1809	98558557-98558805	5	5	5
AFM023xh8	99032436-99032712	1	1/2 *	1
D9S1851	99570721-99571084	2	2	2
XPA (MspI)	100437192-100459691	A	G	G
D9S180	100649474-100649706	1	2	2
D9S6	100931590-100931609	A2	A1	A1
D9S173	101890198-101916471	2	4	2/4 *
D9S176	102058162-102058520	7	3/5 *	5
TGFBR1	103142351-103142640	p.(G52R)	p.(N45S)	p.(R414X)
ALDOB	104182842-104198062	1	1	1/2 *
D9S109	105566169-10556648	2	3	6

The dark shaded boxes indicate contiguous groups of markers defining the shared 4 Mb chromosome 9q22.3 haplotype conserved between families 2,4,5,6,7,8,9,10 and 18 and the 9.9 Mb haplotype shared by families 4–10. The family numbers are from Reference [9]. The lighter shaded box indicates the location of the *TGFBR1* gene within the larger 9.9 Mb haplotype and shows the different *TGFBR1* mutations found in each family or group of families. * For loci where an unfavorable family structure precluded unambiguous identification of which allele was segregating with the condition, both alleles shared by affected family members are shown.
